# Effects of free and encapsulated transglutaminase on the physicochemical, textural, microbial, sensorial, and microstructural properties of white cheese

**DOI:** 10.1002/fsn3.1663

**Published:** 2020-05-21

**Authors:** Fahimeh Razeghi, Sedigheh Yazdanpanah

**Affiliations:** ^1^ Department of Food Science and Technology Kazerun Branch Islamic Azad University Kazerun Iran

**Keywords:** encapsulation, textural and microstructural properties, transglutaminase enzyme, white cheese

## Abstract

In this study, the effect of free and encapsulated transglutaminase (TGase) on physicochemical, textural, microstructural, microbial, and sensorial properties of white cheese was investigated. For this purpose, different types of white cheese incorporated with 20 and 60 ppm free enzyme (F20 and F60) and encapsulated enzyme (E20 and E60) were prepared and then compared with control (C) white cheese without TGase. The results showed that the addition of encapsulated TGase significantly (*p *˂ .05) increased protein and fat content, dry matter, nitrogen recovery, and pH, as well as the production yield of cheeses. The hardness of treated samples was increased during the storage time, while the reverse trend was observed for the control sample. F60 and E60 samples showed more oriented and compact structures compared with other samples. Based on the results of sensory evaluation, E60 sample received the highest taste and flavor scores. Generally, the physicochemical, sensorial, and microstructural properties of white chesses were improved by the presence of encapsulated enzyme in the formulation.

## Graphical abstract

To our best knowledge, the effect of microbial TGase on cheese was only used in free type. But, we think encapsulation of this enzyme have significant effects on the properties of white cheese. Therefore, our main objective was to follow the comparison between free and encapsulated TGase enzyme treatment on cheese. Our final aim was to investigate the effects of free and encapsulated TGase enzyme on the pH, chemical composition, yield, textural, microstructural, microbial, and sensorial properties of the white cheese.

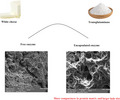

## 1 INTRODUCTION

Transglutaminase (TGase) as a cross‐linking enzyme can create a covalent bond between proteins. This enzyme has high functionality in manufacturing dairy products such as yogurt and cheese (Isleroglu & Turker, 2019; Yokoyama, Nio, & Kikuchi, 2004). TGase catalyzes the intra‐ or inter‐molecular cross‐link formation between ε(γ‐glutamyl) lysine residues using acyl‐transfer reaction. The endoprotein glutamine γ‐carboxamide group acts as an acyl donor and the endoprotein lysine ε‐amino group acts as an acyl acceptor of proteins. TGase can recognize polyamines as an acyl acceptors and so, catalyzes the formation of mono‐ and bis‐(glutaminyl) polyamine derivatives (Sorrentino et al., 2012). Covalent bonding of proteins can improve the techno‐functional and physicochemical properties of food products. In this regard, several researchers have reported that textural properties, water‐holding capacity, nutritional value, film‐forming ability, and film properties of proteins were improved by using TGase (DeJong & Koppelman, 2002; Giosafatto et al., 2018; Sorde & Ananthanarayan, 2019). This technique has been used successfully to modify different types of cereal, meat, bakery, and dairy products. Production of different types of cheese with good textural, sensorial, and microbial properties is one of the main challenges in cheese manufacturing during years. Many attempts have been made by manufacturers to produce cheese with pleasant taste and aroma from liquid milk by different biochemical processing (Litopoulou‐Tzanetaki, 2016; Reis & Malcata, 2011). The rapid growth of the world population has increased the real demand for developing various milk products such as cheese. Therefore, traditional cheese manufacturing has sought new production systems to increase yield production. For this purpose, different types of starter cultures, cheese production machines, as well as refrigeration and pasteurization systems were developed (Ghanimah, Hanafy, Hassanein, & Hashim, 2018; Johnson, 2017). The application of TGase is one of the most promising approaches to increase the production yield of dairy products.

Many researches have been conducted to investigate the effect of direct TGase addition on functional properties of different cheeses including white fresh cheese (García‐Gómez, Vázquez‐Odériz, Muñoz‐Ferreiro, Romero‐Rodríguez, & Vázquez, 2019), semi‐hard cheese (Darnay, Králik, Oros, Koncz, & Firtha, 2017), edam cheesemaking (Aaltonen, Huumonen, & Myllärinen, 2014), trappist cheese (Darnay, Králik, et al., 2017; Darnay, Tóth, et al., 2017), donkey cheese (D'Alessandro, Martemucci, Loizzo, & Faccia, 2019), low‐fat gouda‐like cheese (Ahmed, El‐Nimer, Mostafa, & Omar, 2015), low‐/high‐fat mozzarella cheese (Metwally, El‐Zeini, & Gazar, 2018), and kareish cheese (Darwish & Taher, 2017). It has been reported that the incorporation of encapsulated enzymes can eliminate the problems related to free enzyme addition (Kailasapathy & Lam, 2005). However, there is not any report regarding the encapsulation of TGase for improving techno‐functional properties of cheese. Therefore, the aim of this research was to investigate the effect of encapsulated microbial TGase on the physicochemical, textural, microbial, sensorial, and microstructural properties of white cheese compared with free enzyme addition.

## 2 MATERIALS AND METHODS

### 2.1 Materials

Transglutaminase (TGase) was purchased from Ajinomoto Company. Starter cultures containing *Lactobacillus delbrueckii* ssp., *Bulgaricus*, *Lactococcus lactic* ssp., *cremoris*, *Lactococcus lactis* ssp., *Lactis*, and *Streptococcus thermophilus* were provided from Chr. Hansen Dairy Cultures (RST744). Chymosin as a coagulant was purchased from Ajinomoto Company (standard rennet).

### 2.2 Enzyme encapsulation

Alginate microcapsules were prepared according to the method of Allan‐Wojtas, Hansen, and Paulson (2008). Briefly, alginate powder (10 g/L) was dispersed in deionized distilled water (DDW) and kept 24 hr to completely dissolve the polymer. Then, Tgase was added at different concentrations (20,000 or 60,000 ppm) under stirring for 10 min. The prepared suspension was emulsified by adding into sunflower oil (100 g) containing 5 g/L Tween 80 and then stirred at 900 rpm for 15 min. To initiate the gelation process, 32 ml of an emulsion system containing 62.5 mM CaCl_2_, 60 g oil, and 5 g/L Tween 80 was added into the mixture and mixed for 15 min. Finally, 40 ml CaCl_2_ (0.05 M) was added to form microcapsules. The prepared microcapsules were removed by filter paper.

### 2.3 Cheesemaking

For making white cheese, at first, milk was pasteurized at 65°C for 5 min. Then, the pasteurized milk was cooled to 35°C and the starter culture and CaCl_2_ were added at concentrations of 0.04 g/L kg and 0.1 g/L milk, respectively. After 30 min of incubation at 35°C, rennet (0.025 g/kg) and TGase (20–60 ppm) were added. For two experimental cheeses, TGase was directly used in the free form (F20 and F60) and the other two samples were formulated with encapsulated Tgase in alginate microcapsules. For the coagulation of milk, samples were incubated for 45 min 35°C. Coagulation was started after 10 min, and milk was gelled after 45 min. The prepared samples were cut in 1 cm^3^ cubes and then kept constant for 20 min. Then, they were transferred into a cloth and pressed at 25°C for 2.5 hr. Afterward, cheese samples were removed and cut to 4 × 6 × 6 cm^3^ cubes. The cheese cubes were placed in sodium chloride solution (13% w/w) for 19 hr at 23°C. After the ripening process for 60 days at 5–6°C, different properties of white cheese samples were investigated (Hayaloglu, Guven, & Fox, 2002). The control cheeses were produced in similar ways without TGase addition.

### 2.4 pH measurement

Ten gram of each sample was homogenized with 90 g DDW, and the pH was measured using a digital pH meter (Suntex TS‐1) (Morin‐Sardin, Rigalma, Coroller, Jany, & Coton, 2016).

### 2.5 Chemical composition

Fat content, dry matter, protein content, and salt content were determined according to AOAC (Chemists & Horwitz, 1990). Nitrogen recovery from cheese was determined according to the method of Johnson, Chen, and Jaeggi (2001).

### 2.6 The yield of cheese production

To obtain the yield of cheese production, the initial weight of prepared cheese (after 1 day of storage at room temperature) was divided into the weight of the used milk (Martí‐De Olives, Peris, & Molina, 2019).

### 2.7 Textural analysis

Textural analysis of samples was performed by double compression test (TPA) using Texture Analyzer (CT34500) to obtain different parameters including hardness, springiness, cohesiveness, gumminess, adhesiveness, and chewiness. Cheese samples were cut to cylinder shape with 15 mm diameter and 15 mm height and kept at 25°C for 0.5 hr. The compression height and test speed were 66.6% of the initial height and 0.5 mm/s, respectively. TA25/1000 probe with 5 g trigger force was used for measurement. Textural parameters were analyzed using the Exponent Lite software (Stable Microsystems) from the obtained force versus time curves (Brighenti, Govindasamy‐Lucey, Jaeggi, Johnson, & Lucey, 2018).

### 2.8 Scanning electron microscopy (SEM)

The microstructure of alginate microcapsules and cheese samples were observed with scanning electron microscopy (SEM) using the technique described by Rahimi, Khosrowshahi, Madadlou, and Aziznia (2007) with some modifications. A slice of chesses sample was separated and fixed by glutaraldehyde (2.5%, w/w) for 180 min. The prepared sample was washed by DDW for 6 min. Ethanol (30%, 50%, 75%, and 99%) was used for dehydration of wet samples after washing with water for 30 min. Then, chloroform was used for the extraction of lipids (twice times, each time 15 min). The small pieces of dried and defatted samples were frozen by liquid nitrogen, then coated with a thin layer of gold for 15 min in a sputter‐coater (Desk Sputter CoaterDSR1, Nanostructural Coating Co.). Sample images were taken by a scanning electron microscope (TESCAN vega3) operated at an accelerated voltage of 15.0 kV.

### 2.9 Microbial count

Coliform, *E. coli*, yeast and mold, Salmonella and Staphylococcus were determined based on the ISIRI standard method.

### 2.10 Sensorial analysis

Twenty panelists (Semi trained students of food science and technology department of Azad University of kazerun, 10 males and 10 females) in the range of 23–33 years old evaluated the sensorial attributes of cheese samples including taste, color, and odor (Pino et al., 2018). Samples were studied by a 5‐point hedonic scale (1 = do not like, 5 = liked). Cheese samples were placed in airtight plastic containers and kept at 25°C for 120 min before analysis. To cleanse the palate, water was used by panelists. The sensorial evaluation was conducted every 20 days during 60 days storage period.

### 2.11 Statistical analysis

Results were analyzed by SPSS V. 19.0.0. Significance differences between the mean values were determined by one‐way ANOVA variance analysis. Duncan's multiple range test at *p* < .05 was used for comparison of means.

## 3 RESULTS AND DISCUSSION

### 3.1 Microstructure of microcapsules

**FIGURE 1:**
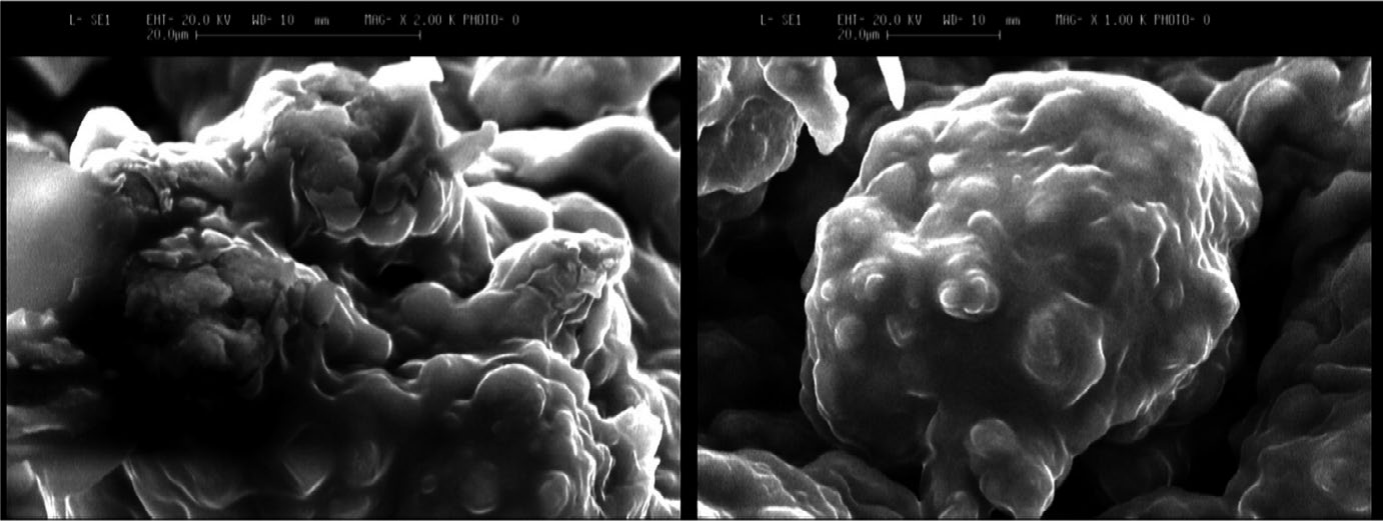
Scanning electron microscopy (SEM) of calcium alginate microcapsules. (a) Microcapsule loaded with 20 ppm, (b) microcapsule loaded with 60 ppm

The microstructure of microcapsules containing TGase was shown in Figure 1. Microcapsules were not spherical, and the size of microcapsules was around 50 µm. The same results were reported by Ahari, Fahimdanesh, Khosravi Zanjani, Anvar, and Shokri (2012).

### 3.2 Cheese production yield

The effect of free and encapsulated TGase on the yield of cheese production is reported in Table 1. Generally, production yield was increased by the addition of TGase in both free and encapsulated forms. Therefore, the highest and the lowest cheese production yield were related to E60 sample (8.75%) and the control sample (7.30%), respectively. This increase was due to the increase in the protein and moisture content of cheese. In fact, TGase increased the protein content of samples by providing covalent bonding between the milk protein molecules. The same result was reported by García‐Gómez et al. (2019) and Bönisch, Heidebach, and Kulozik (2008). Moreover, the increase in the production yield may be due to the cross‐linking between hydrophilic parts of κ‐casein during coagulation of milk protein (Karzan, Nawal, & Ashna, 2016). Similarly, Bönisch et al. (2008) reported that the increase of serum binding in gel structure increased the production yield, which was due to additional covalent bonds of casein in the gel structure.

**TABLE 1:** Yield of samples

	Control	Free enzyme		Encapsulated enzyme	
		20	60	20	60
Yield	7.30 ± 0.14d	8.50 ± 0.14c	9.05 ± 0.21a	8.40 ± 0.14c	8.75 ± 0.07b

Note

Data represent mean ± standard deviation of three independent repeats. Different lowercase letters in each row indicate significant differences (*p* < .05).

### 3.3 Chemical composition

Table 2 shows the effect of free and encapsulated TGase on the cheese protein content. The presence of enzyme, either free enzyme or encapsulated form, increased the protein content significantly (*p* ˂ .05). Increasing the concentration of TGase led to a further increase in protein content. Therefore, the cheese sample containing 60 ppm of encapsulated enzyme and control sample had the highest and lowest protein content, respectively. TGase enzyme can create covalent bonds between glutamine and lysine. It can also create cross‐link between different forms of casein and bind casein micelles together. Beta‐lactoglobulin and α‐lactalbumin consider as good substrates for TGase (Aaltonen et al., 2014). Storage time had no significant effect on protein content. The results of fat content (Table 2) showed that the addition of 60 ppm TGase increased the fat content significantly; however, storage time had no significant effect. This may be related to the extra fat added to the cheese formulation due to the preparation of alginate capsules in an oil emulsification process (Kailasapathy & Lam, 2005). TGase can affect this parameter by changing the gel structure. Darnay, Králik, et al. (2017) and Darnay, Tóth, et al. (2017) also reported that by addition TGase to semi‐hard cheese, fat content significantly (*p* ˂ .05) increased. In agreement with the obtained results for protein and fat content, the dry matter was increased as affected by TGase addition. Karzan et al. (2016) also reported that the modified cheese with TGase had a higher dry matter. The result of nitrogen recovery showed that the addition of enzyme had a significant effect on the nitrogen recovery and sample containing 60 ppm enzyme had the highest value. Johnson et al. (2001) reported the nitrogen recovery of their sample was around 74%. The results of salt content showed that samples containing encapsulated enzyme had the highest salt concentration, which is due to using of salts in preparing alginate microcapsule as carrier of TGase.

**TABLE 2:** Protein, fat, dry matter, nitrogen recovery, and salt content of cheese treated with free and encapsulated transglutaminase

	Storage (day)			
	0	20	40	60
Protein				
C	13.13 ± 0.01Cb	13.17 ± 0.01Ca	13.19 ± 0.01Ca	13.19 ± 0.02Ca
F20	16.58 ± 0.05Ba	16.55 ± 0.05Ba	16.50 ± 0.01Ba	16.50 ± 0.05Ba
F60	17.05 ± 0.05Ba	16.93 ± 0.05Ba	16.95 ± 0.05Ba	17.03 ± 0.05Ba
E20	16.90 ± 0.07Ba	16.94 ± 0.07Ba	17.01 ± 0.01Ba	16.95 ± 0.07Ba
E60	17.21 ± 0.02Aa	17.19 ± 0.02Aa	17.21 ± 0.02Aa	17.18 ± 0.02Aa
Fat				
C	11.20 ± 0.01Ba	11.22 ± 0.03Ba	11.22 ± 0.01Ba	11.23 ± 0.09Ba
F20	11.26 ± 0.05Ba	11.25 ± 0.05Ba	11.26 ± 0.05Ba	11.24 ± 0.05Ba
F60	11.42 ± 0.05Aa	11.33 ± 0.05Aa	11.37 ± 0.05Aa	11.41 ± 0.06Aa
E20	11.22 ± 0.07Ba	11.22 ± 0.07Ba	11.23 ± 0.07Ba	11.22 ± 0.07Ba
E60	11.34 ± 0.02Aa	11.37 ± 0.02Aa	11.30 ± 0.02Aa	11.34 ± 0.02Aa
Dry matter				
C	27.27 ± 0.26Ea	27.69 ± 0.08Ea	27.58 ± 0.14Ea	27.71 ± 0.04Ea
F20	29.61 ± 0.05Da	29.40 ± 0.08Da	29.37 ± 0.07Da	29.41 ± 0.03Da
F60	33.66 ± 0.05Ba	33.68 ± 0.06Ba	33.59 ± 0.03Ba	33.60 ± 0.06Ba
E20	32.62 ± 0.13Ca	32.47 ± 0.05Ca	32.62 ± 0.12Ca	32.22 ± 0.19Ca
E60	35.22 ± 0.02Aa	35.37 ± 0.07Aa	35.30 ± 0.02Aa	35.34 ± 0.09A
Nitrogen recovery				
C	60.65 ± 0.68Ca	60.63 ± 0.55Ca	60.66 ± 0.38Ca	60.59 ± 0.13Ca
F20	64.65 ± 0.74Ba	64.70 ± 0.11Ba	64.65 ± 0.49Ba	64.60 ± 0.44Ba
F60	67.67 ± 0.62Aa	67.59 ± 0.39Aa	67.67 ± 0.17Aa	67.63 ± 0.36Aa
E20	64.23 ± 0.95Ba	64.92 ± 0.34Ba	64.69 ± 0.51Ba	64.81 ± 0.29Ba
E60	66.61 ± 0.74Aa	66.43 ± 0.43Aa	66.38 ± 0.33Aa	66.15 ± 0.72Aa
Salt				
C	5.47 ± 0.02Ca	5.49 ± 0.02Ca	5.48 ± 0.02Ca	5.49 ± 0.02Ca
F20	5.55 ± 0.02Ba	5.57 ± 0.02Ba	5.57 ± 0.02Ba	5.60 ± 0.02Ba
F60	5.45 ± 0.01Ca	5.49 ± 0.01Ca	5.48 ± 0.01Ca	5.50 ± 0.01Ca
E20	5.62 ± 0.02Aa	5.63 ± 0.02Aa	5.65 ± 0.02Aa	5.69 ± 0.02Aa
E60	5.62 ± 0.07Aa	5.66 ± 0.07Aa	5.69 ± 0.05Aa	5.69 ± 0.01Aa

Note

Data represent mean ± standard deviation of three independent repeats. Different capital letters in each column and lowercase ones in each row indicate significant differences (*p* < .05).

### 3.4 pH

The pH of treated samples with free and encapsulated TGase during storage is reported in Table 3. The results showed that the control sample had the maximum changes during storage and sample treated with 60 ppm of the encapsulated enzyme had the minimum changes. This result probably was related to the hardness of structure. Application of TGase resulted in a harder structure due to the covalent cross‐linking of cheese proteins (Heidebach, Först, & Kulozik, 2009). It was also observed that the pH of all samples significantly decreased during the storage time due to the conversion of lactose to lactic acid by microorganisms (D'Alessandro et al., 2019) and production of free fatty acids through lipid oxidation. Similar results regarding the effect of oxidation and lactic acid production by microorganisms in reducing pH were reported by Darwish and Taher (2017) and D'Alessandro et al. (2019).

**TABLE 3:** pH of cheese treated with free and encapsulated transglutaminase

	Storage (day)			
	0	20	40	60
C	4.58 ± 0.07Aa	4.51 ± 0.02Ba	4.31 ± 0.01Db	4.18 ± 0.01Dc
F20	4.56 ± 0.01Aa	4.56 ± 0.02Aa	4.52 ± 0.01Ab	4.29 ± 0.02Bc
F60	4.65 ± 0.07Aa	4.55 ± 0.02Ab	4.48 ± 0.01Bc	4.32 ± 0.01Bd
E20	4.66 ± 0.07Aa	4.54 ± 0.02Ab	4.38 ± 0.01Cc	4.24 ± 0.01Cd
E60	4.58 ± 0.07Aa	4.57 ± 0.01Aa	4.53 ± 0.07Aa	4.39 ± 0.01Ab

Note

Data represent mean ± standard deviation of three independent repeats. Different capital letters in each column and lowercase ones in each row indicate significant differences (*p* < .05).

### 3.5 Textural properties

Textural properties including hardness, springiness, cohesiveness, gumminess, adhesiveness, and chewiness of cheese samples treated with free and encapsulated TGase are shown in Table 4. In the control sample, the hardness value was decreased significantly during storage time. In contrast, those samples treated with TGase showed a significant increase in hardness at the same time. Gumminess and chewiness parameters showed similar trends. Aaltonen et al. (2014) reported that samples treated with TGase had the most changes in their water content and hardness due to covalent bonds formed by TGase. Darnay, Králik, et al. (2017)) and Darnay, Tóth, et al. (2017) reported the same trend about the effect of TGase on the hardness parameter.

**TABLE 4:** Textural properties of cheese treated with free and encapsulated transglutaminase

	Storage (day)			
	0	20	40	60
Hardness (g)				
C	3,278.00 ± 94.75Aa	2,152.25 ± 45.60Ab	1,845.20 ± 48.22Bc	1,202.05 ± 13.51Cd
F20	1,460.75 ± 27.93Bc	1,442.85 ± 54.94Bc	1,657.20 ± 50.62Cb	2,162.10 ± 39.17Ba
F60	1,202.25 ± 13.78Cd	2,190.00 ± 18.38Ac	3,250.70 ± 54.16Ab	3,875.55 ± 47.72Aa
E20	1,479.60 ± 11.87Bc	1,369.85 ± 30.61Bc	1,561.90 ± 42.00Cb	2,161.15 ± 25.10Ba
E60	1,222.80 ± 16.54Cd	2,167.35 ± 62.29Ac	3,249.65 ± 24.67Ab	3,782.60 ± 57.41Aa
Springiness (mm)				
C	8.87 ± 0.07Bb	9.08 ± 0.10Ca	9.13 ± 0.02Ba	9.15 ± 0.02Ca
F20	9.17 ± 0.04Aa	9.14 ± 0.03Ba	9.18 ± 0.02Ba	9.21 ± 0.02Ba
F60	8.76 ± 0.13Bd	9.38 ± 0.04Ac	9.46 ± 0.01Ab	9.55 ± 0.03Aa
E20	9.18 ± 0.01Aa	9.14 ± 0.01Bb	9.14 ± 0.01Bb	9.20 ± 0.02Ba
E60	8.73 ± 0.01Bd	9.31 ± 0.02Ac	9.44 ± 0.02Ab	9.54 ± 0.01Aa
Cohesiveness				
C	0.67 ± 0.01Ac	0.77 ± 0.02Bb	0.81 ± 0.02Bb	0.86 ± 0.01Ba
F20	0.68 ± 0.01Ab	0.84 ± 0.02Aa	0.87 ± 0.01Aa	0.89 ± 0.02Aa
F60	0.73 ± 0.01Ac	0.86 ± 0.02Ab	0.89 ± 0.01Ab	0.94 ± 0.01Aa
E20	0.64 ± 0.01Bb	0.83 ± 0.01Aa	0.86 ± 0.01Aa	0.86 ± 0.02Ba
E60	0.70 ± 0.01Ab	0.86 ± 0.012a	0.87 ± 0.01Aa	0.90 ± 0.01Aa
Gumminess (g)				
C	2,231.20 ± 42.70Aa	1,650.20 ± 41.15Ab	1,408.70 ± 31.96Bc	1,269.10 ± 31.53Bd
F20	1,305.90 ± 35.21Ba	1,200.50 ± 43.84Bb	1,291.65 ± 18.17Ba	1,363.50 ± 26.30Ba
F60	869.45 ± 3.74Cd	1,791.16 ± 39.51Ac	2,112.40 ± 15.41Ab	2,532.40 ± 12.40Aa
E20	1,307.30 ± 19.65Ba	1,184.50 ± 35.77Bb	1,281.30 ± 53.59Ba	1,351.05 ± 2.33Ba
E60	861.15 ± 11.24Cd	1,797.75 ± 21.99Ac	2,122.35 ± 12.79Ab	2,548.55 ± 16.19Aa
Adhesiveness (mj)				
C	3.28 ± 0.04Aa	0.52 ± 0.01Ab	0.43 ± 0.02Ac	0.30 ± 0.04Ad
F20	1.07 ± 0.07Ba	0.21 ± 0.01Bb	0.18 ± 0.01Bc	0.11 ± 0.01Bd
F60	0.28 ± 0.02Ca	0.08 ± 0.01Cb	0.02 ± 0.01Cc	0.01 ± 0.01Cc
E20	1.10 ± 0.01Ba	0.19 ± 0.01Bb	0.16 ± 0.01Bc	0.11 ± 0.02Bd
E60	0.29 ± 0.02Ca	0.05 ± 0.01Cb	0.03 ± 0.01Cc	0.01 ± 0.01Cc
Chewiness (mj)				
C	194.57 ± 4.90Aa	155.32 ± 6.32Ab	120.35 ± 1.76Bc	95.85 ± 3.74Cd
F20	119.10 ± 1.54Ba	104.20 ± 0.56Bc	109.95 ± 2.19Bb	91.45 ± 3.1BCd
F60	72.80 ± 3.54Cd	164.95 ± 2.61Ac	191.25 ± 3.04Ab	220.40 ± 1.13Aa
E20	118.80 ± 0.98Ba	101.05 ± 0.21Bc	108.65 ± 2.19Bb	84.85 ± 0.49Cd
E60	72.70 ± 2.12Cd	160.95 ± 0.21Ac	188.80 ± 1.97Ab	219.25 ± 1.34Aa

Note

Data represent mean ± standard deviation of three independent repeats. Different capital letters in each column and lowercase ones in each row indicate significant differences (*p* < .05).

The results of springiness and cohesiveness showed that these parameters significantly increased during the storage time and samples containing 60 ppm of enzyme had the highest springiness and cohesiveness values. The results of adhesiveness showed that this parameter was significantly reduced during the storage time and samples containing 60 ppm of enzyme had the lowest adhesiveness. Ahmed et al. (2015) observed that TGase could improve the emulsion and foam‐forming abilities, water‐holding capacity, and viscosity in low fat cheese. García‐Gómez et al. (2019) reported that the addition of TGase to cheese sample prepared with chymosin significantly enhanced springiness and chewiness by 9% and 19%, respectively. They also reported that cohesiveness of the sample treated with TGase was greater than control. These findings were similar to the results of other researchers (Özer, Hayaloglu, Yaman, Gürsoy, & Şener, 2013).

### 3.6 Sensorial properties

**FIGURE 2:**
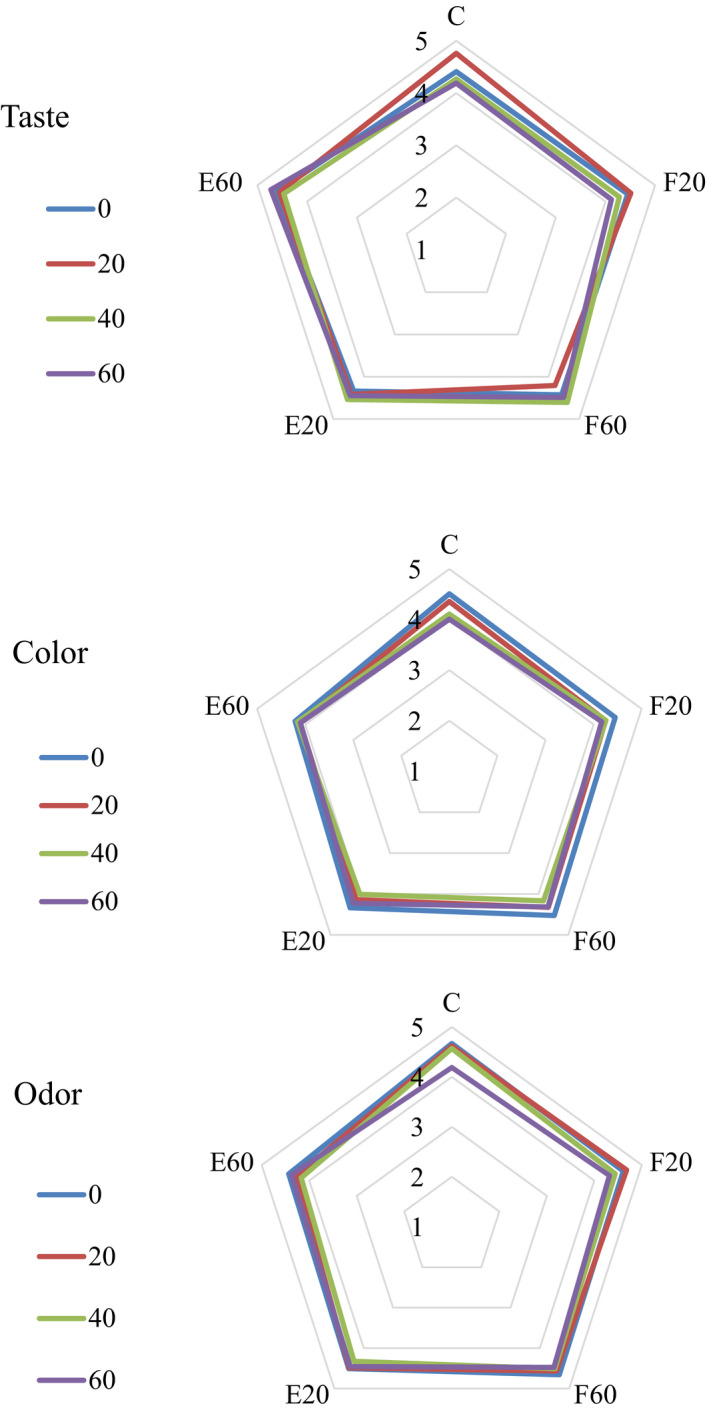
Sensorial properties of cheese treated with free and encapsulated transglutaminase

Figure 2 presents the effects of free and encapsulated TGase on sensorial properties including taste, color, and odor. The results of taste evaluation showed that the most acceptable taste attributed to the sample incorporated with 60 ppm encapsulated enzyme. While the sample prepared with 60 ppm of free enzyme obtained the highest scores for color and odor. Karzan et al. (2016) studied the effects of TGase on the sensorial properties of goat's cheese. They reported that the color, flavor, and taste of treated samples with TGase were significantly better than control sample. Darwish and Taher (2017) also studied the effects of TGase on the sensorial properties of Kareish cheese. Similarly, they reported the flavor of treated samples with TGase was significantly better than control sample. Moreover, similar results on the sensorial properties of UF‐white soft cheese modified by TGase was reported by Ibrahim et al. (2017).

### 3.7 Microbial properties

Evaluation of microbial properties of samples showed that yeast and mold, coliform, *E. coli*, salmonella, and staphylococcus bacteria counts were in the permitted ISIRI standard range. The initial count of milk, processing temperature, processing time, and production condition can affect the microbial count of final products. Temperature, production of acid lactic and H^+^, as well as the increase in dry matter and chloride content are the main factor in the viability of microorganism in the cheese (Caridi, Micari, Caparra, Cufari, & Sarullo, 2003).

### 3.8 Microstructural properties

**FIGURE 3:**
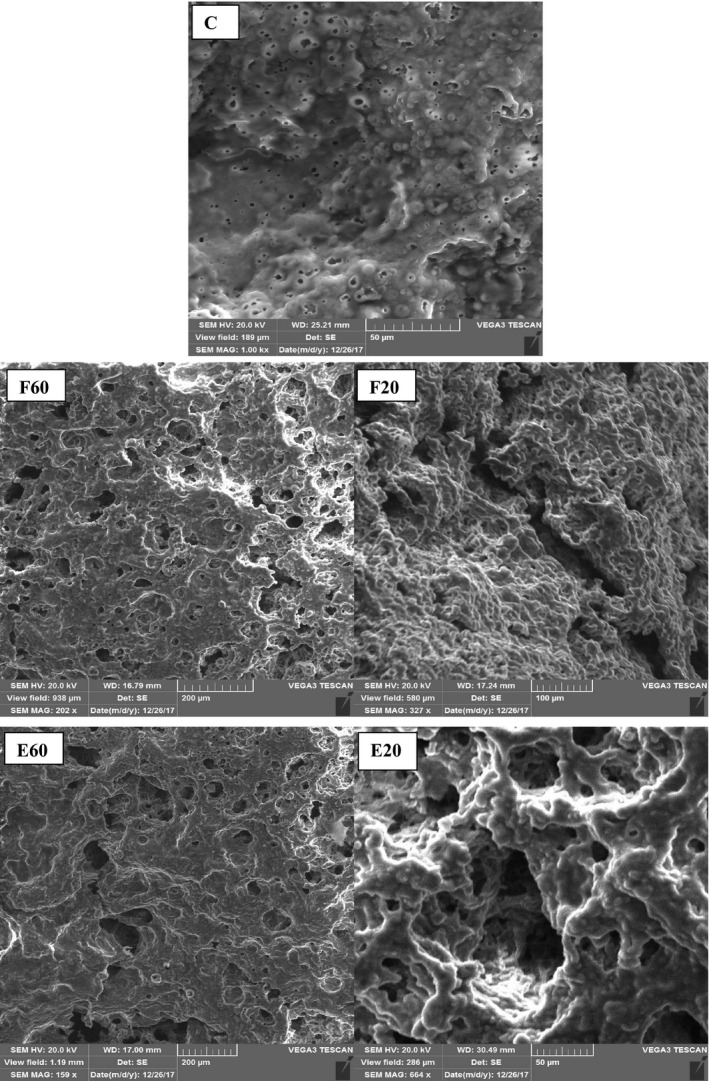
Scanning electron microscopy (SEM) of cheeses. (C) Control sample without Tgase, (F60) sample containing 60 ppm free enzyme, (F20) sample containing 20 ppm free enzyme, (E60) sample containing 60 ppm encapsulated enzyme, and (E20) sample containing 20 ppm encapsulated enzyme

Control sample had a uniform surface with low porosity. The addition of free TGase enzyme created a dense microstructure in chesses. Samples containing free enzyme showed higher porosity in comparison with control. The addition of encapsulated enzyme had more effects on the structure and resulted in more compactness in protein matrix. The size of the holes in cheese samples containing encapsulated enzyme was higher than that of samples containing free enzyme (Figure 3). Similar result was observed by Metwally et al. (2018).

## 4 CONCLUSION

This research is the first report regarding the effect of different concentrations of free and encapsulated TGase on techno‐functional and textural properties of white cheese. The production yield, protein content, fat content, dry matter, nitrogen recovery, and pH of samples increased by addition of TGase. Textural properties were also improved after treating with TGase. Sensorial properties showed that samples prepared with 60 ppm of enzyme had the highest scores. However, sample containing 60 ppm of encapsulated enzyme received the highest acceptability. This study has represented that application of encapsulated TGase into cheese matrix is an effective tool to create acceptable texture and enhance production yield in cheese products.

## CONFLICT OF INTEREST

The authors declare that they do not have any conflict of interest.

## ETHICAL APPROVAL

Production of this product was done in sterile condition and before consumption by panelist, microbial analysis was evaluated. Also sensorial evaluation in the supervision of Dr. Yazdanpanah in test panelist room was done.

## INFORMED CONSENT

Written informed consent was obtained from all study participants.
